# The role of synergistic interplay among proactive personality, leader creativity expectations, and role clarity in stimulating employee creativity

**DOI:** 10.3389/fpsyg.2022.699411

**Published:** 2022-07-22

**Authors:** Xiaohong Wang, Meng Wang, Feng Xu

**Affiliations:** ^1^School of Management, Harbin Institute of Technology, Harbin, China; ^2^School of Humanities, Social Sciences & Law, Harbin Institute of Technology, Harbin, China

**Keywords:** proactive personality, leader creativity expectations, employee creativity, role clarity, role theory, Pygmalion effect of creativity

## Abstract

This study investigates the interplay among proactive personality, leader creativity expectations, and role clarity in stimulating employee creativity based on the theoretical frameworks of role theory. Questionnaires were distributed to obtain 290 leader-employee dyads from China to examine hypotheses *via* conditional process analysis. The results show that proactive personality has a positive effect on employee creativity, leader creativity expectations did not play a significant moderating role on the relationship between proactive personality and employee creativity. The interaction between leader creativity expectations and role clarity has a significant moderating effect on the relationship between proactive personality and employee creativity. These findings are discussed in terms of their theoretical and practical significance.

## Introduction

As organizations seek to effectively navigate today’s highly competitive market, they need require employees creatively solve various problems throughout the workplace (Xu and Wang, 2019). Academics broadly define “creativity” as “the ability to produce innovative and practical ideas” (Amabile, 1988). Factors influencing creativity in this context include organizational culture, job design, leadership, human resource management, and personality characteristics (Gong et al., 2009; Zhang et al., 2020, 2022). Individual factors are generally regarded as the main source of employee creativity (Shalley et al., 2004), particularly personality characteristics that may be deep, intrinsic motivations for creative work (Zhang et al., 2019).

Research have shown that proactive individuals tend to behave under stronger intrinsic motivations than those who are less proactive, and that creative behaviors can be considered a kind of “proactive action” (Ng and Feldman, 2013; Kim, 2019). Recent studies have shown that a proactive personality is extremely important in terms of creativity, and proactive personality can be a proxy indicator of creativity among an individual’s personal characteristics (Tai and Mai, 2016; Kim, 2019). Individuals with proactive personality are also predicted to perform better than those with the “Big Five” in many work situations (Marinova et al., 2015).

Although individual factors are the primary, decisive source of creativity (Shalley et al., 2004; Carmeli and Schaubroeck, 2007), a given individual may newly develop creativity due to immersion in an environment that encourages or stimulates creative behavior (Wang et al., 2022). For example, a typical case is the Pygmalion effect (Tierney and Farmer, 2004). Despite extant studies offer valuable contributions to the literature regarding contextual factors such as work environment, leadership, and colleague relationships that may occur simultaneously and affect the relationship between proactive personality and individual creativity (Shalley et al., 2004; Kim et al., 2010; Wang et al., 2014; Chen et al., 2016; Kim, 2019; Mubarak et al., 2021), the effect of role expectations on the relationship between proactive personality and creativity is not yet well understood.

One with a proactive personality has a “stable tendency that is relatively free of environmental constraints and can influence the surrounding environment by taking active actions” (Bateman and Crant, 1993). This definition of the proactive personality, however, not necessarily includes self-orientation, namely, the expectations of creativity under which the individual operates. Such expectations are important to consider, however, as the orientation of individual behavior is shaped largely by the environment. As one of the key environmental factors in the workplace, the leadership factor is highly influential (Joo et al., 2013; Chen and Hou, 2016; Hughes et al., 2018). Scholars have previously explored the influence of leaders’ expectations on employees’ creative work (Qu et al., 2015a). Leaders’ expectations of creativity can be considered as an external motivator for promoting (or hindering) their employees’ independent innovation abilities in different situations (Zhao and Guo, 2019). Based on the theory of role theory (Anglin et al., 2022), the interaction between individual internal and contextual factors can deepen our understanding of the Pygmalion effect of creativity.

In this study, we first focus on the possible influence of leader creativity expectations by determining whether such influence plays a moderating role between proactive personality and employee creativity. Previous researchers have tended to focus on the manner in which leaders shape their employees’ creative behavior by their expectations, but generally ignore the response of employees to such creativity expectations (Qu et al., 2015b; Jiang and Gu, 2017; Xu and Wang, 2019). However, in practice, employees tend to respond strongly to support and encouragement from others. Their work—including creative work—is also influenced by the roles they are given by their supervisors (Eden, 1992; Jada et al., 2019). Considering only the role expectations placed on employees does not comprehensively reveal the potential to improve their creative performance (Väänänen et al., 2005).

Role indicates the expectations and desires of the individual and organization from each other (Adil et al., 2021). According to role theory (Anglin et al., 2022), we instead focus on the bidirectional nature of role expectations in regard to employee creativity. The content of one’s work, beyond leaders’ expectations, is also an important factor in the environment in which that work takes place. The influence of work content on employee creativity has been given relatively little research attention. Creativity is a high-level cognitive process; producing innovative, high-quality solutions necessitates “creative thinking” (Xu and Wang, 2019). Novelty, complexity, and ambiguity act differently on different people as they attempt to problem-solve. Problems requiring creative thinking in the field of physical sciences markedly differ from those requiring creative thinking in the humanities field (Baer, 2003). Role clarity indicates the extent to which employees acquire and understand the information or data required to complete their work (Kelly and Richard, 1980; Adil et al., 2021). Employees who are short on role clarity often cannot maintain progress or positivity; they tend to not feel encouraged or supported by their superiors, which is a known predictive factor of deviant behavior (Judge et al., 2006; Bang et al., 2022; Orgambídez et al., 2022).

Sawyer (1992) defines “role clarity” from two dimensions. The first is “goal clarity,” namely, the degree to which employees clearly understand the purposes of their work and the responsibilities relevant to those purposes. The second is “process clarity,” which refers to the employees’ understanding of the operations necessary to achieve these goals. Employees with higher role clarity understand the expectations placed upon them as well as the methods and processes they should adopt to achieve their goals. A clearer understanding of the core aspects of their work allows employees to communicate effectively with leaders to achieve goals as a team. Individuals’ perceptions of their job responsibilities may determine the extent to which they understand the creativity expected of them by their leaders.

**Figure 1:**
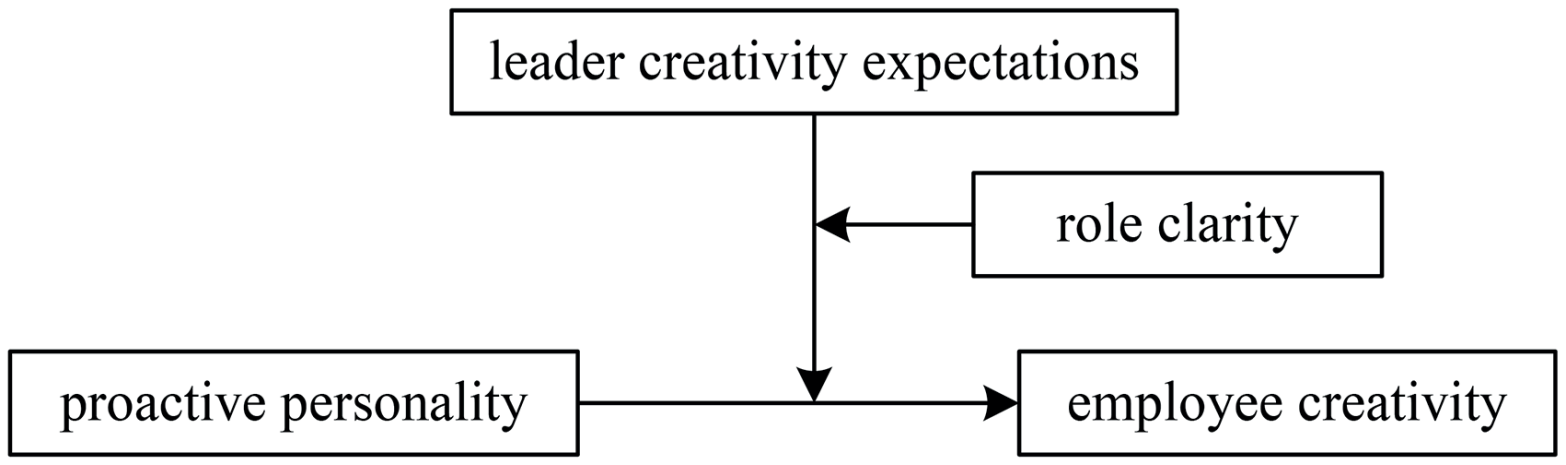
Hypothesized model.

This research aims to answer a theoretically relevant question of when and how interactions of three types of antecedents (proactive personality, leader creativity expectations, and role clarity) lead to varying levels of employee creativity. While investigating the influence of proactive personality on employee creativity, we explore the bidirectional support of leader creativity expectations and role clarity. By simultaneously considering the influence of both leader creativity expectations and employee role clarity, we seek to investigate the role proactive personality has in shaping creative performance based on a role shaping perspective. Our hypothesized model is shown in Figure 1.

## Literature review and hypothesis statements

### Proactive personality and employee creativity

Proactive behaviors mainly include prediction, change orientation, and self-motivation (McCormick et al., 2019). Innovation behavior is closely related to proactivity. A proactive personality is considered an important antecedent of a variety of individually proactive behaviors. Individuals with proactive personalities are usually more sensitive in their work than non-proactive individuals; they more actively seize work-related opportunities (Alikaj et al., 2021). Positive, proactive behaviors tend to be driven by high intrinsic motivations to obtain praise, encouragement, or promotions (Antonacopoulou, 2000; Tolentino et al., 2014).

In general, there is a positive correlation between proactive personality, self-learning orientation, and self-efficacy of learning in employees. Proactive employees are more likely to improve their own work-related abilities and gain knowledge and skills related to their work field on their own volition, thereby showing a stronger tendency to innovate (Kim, 2019). Employees with proactive personalities tend to be self-created, future-oriented, and transformation-oriented, which may also support the generation of creativity (Parker et al., 2010; Jiang and Gu, 2015). Studies have shown that employees with proactive personalities manage pressure effectively, allowing them to utilize the knowledge and skills they have gained to creatively solve problems at work (Vignoli and Depolo, 2019; Mubarak et al., 2021).

The active expression of personal ideas is another important characteristic related to a proactive personality. To this effect, proactive employees may have stronger communication abilities than non-proactive employees. By communicating with colleagues, these employees can gain support for their ideas among both peers and higher-level individuals, accelerating the creativity of the entire organization (Thompson, 2005). As per the social exchange theory, proactive employees may become more efficient after other individuals show trust, encouragement, or other actively supportive feedback, thereby actively engaging in further proactive behaviors. This feedback process continually improves creativity (Gong et al., 2012). Employees with low proactivity, conversely, may be more inclined to make passive responses to workplace situations, be less likely to gain knowledge and skills in the work field on their own, and struggle to identify opportunities in the workplace, thus experiencing less motivation for independent creative behaviors (Mubarak et al., 2021). Although the relationship between proactive personality and employee creativity is not the focus of this study, we still propose the following hypothesis for the sake of research integrity:

*H1*: Proactive personality positively related to employee creativity.

### Leader expectations for creativity

Although individual personality characteristics are the main source of and key factor in creativity, a proactive personality does not necessarily relate to the expectations for creative behaviors placed upon the individual by his or her supervisor. The behavior orientation of subordinates largely depends on the influence of leaders (Xu and Wang, 2019). Further, leaders are often considered to be representatives of organizations (Bysted and Jespersen, 2014). Subordinates tend to closely focus on the traits and behaviors of their leaders (Zhang et al., 2020).

Leaders tend to be the initiators of innovation (Xu and Wang, 2019). Innovation invariably accompanies risk. Risk, and other changes brought on by employees’ creative ideas and behaviors, can challenge established work objectives, working methods, task relationships, and informal norms (Bysted and Jespersen, 2014). These challenges create turbulence and place pressure on executive leadership. In this sense, innovation is driven from the top down; employee creativity thus requires specific signals sent by leaders (Bysted and Jespersen, 2014). When discussing the manner in which leadership behavior motivates innovation, it is important to emphasize that for the vast majority of employees, innovation is deemed as an extra-role behavior (Qu et al., 2015b). As behavioral practices within the organization usually refer to the successful experiences of the past, employees tend to use known solutions to solve similar problems at work rather than seeking new solutions (Ford, 1996). Creative problem-solving carries higher risk, as it requires employees to believe that innovative, new behaviors will be successful. In the absence of this belief, employees will not take the risks to perform beyond their own duties. The attitude of leadership toward innovation is crucial, as employees may depend on top-down motivations for creativity (Xu and Wang, 2019).

Under the “Pygmalion effect,” an individual’s expectations or predictions based on their perception of a certain situation allow them to adapt. Thus, a leader’s expectations are likely to facilitate followers generate creativity. Role expectations can clearly indicate the work that employees should undertake, which plays an important role in shaping role behavior (Dierdorff and Morgeson, 2007). As an important external motivation, leader creativity expectations can significantly influence creative behavior (Qu et al., 2015b; Jiang and Gu, 2017; Nabi et al., 2022). Leaders have an important legal position in the organization as well as the control over their employees’ work, including task allocation, performance appraisals, salaries, personnel transfers, and promotions (Xu and Wang, 2019). Employees thus observe and deduce the expectations of their leaders and respond in kind. When leaders prioritize creativity, and set clear expectations for creativity, their followers are more likely to be creative.

Employees with proactive personalities tend to excel at finding opportunities to enhance their current work, and to take positive actions to continuously influence their surrounding environment (Bateman and Crant, 1993; Crant, 2000). Innovative or creative activities may be more time-consuming and riskier than existing practices (Dewett, 2006), so leaders with creativity expectations should provide greater external support. Creative work also requires high-level cognitive ability and occupies a large amount of resources (Shalley et al., 2009), so leaders with creativity expectations should be mindful of this and timely replenish the resources consumed. Leader creativity expectations can indicate the value of creativity and encouragement of creativity to a certain extent within their organization (Jaussi and Dionne, 2003; Carmeli and Schaubroeck, 2007; Kark and Carmeli, 2009). Further, in individuals with higher self-efficacy (Zhao and Guo, 2019), their perceived leader creativity expectations are directly proportional to their engagement with creative work. When employees’ high proactive personalities sense that their leaders expect them to be creative, they will search out and seize opportunities to do so. However, not all employees sense such implications. Those with non-proactive personalities may regard the leader’s creativity expectations as external pressure under which they will adopt passive behavior or rebellious attitudes.

Based on the above theoretical analysis and empirical evidence, we find that when the leaders clearly express expectations for innovation, proactive employees will see these expectations as opportunities to engage in creative work. They will respond autonomously, generating creativity that is then fed back into the organization in a cyclical manner. We developed the following hypothesis accordingly:

*H2*: Leader creativity expectations positively moderate the relationship between proactive personality and employee creativity.

### Role clarity

Early researchers tended to emphasize the positive role of leaders in shaping employee behavior, where subordinates are generally conceptualized as passive recipients (Väänänen et al., 2005). The “Pygmalion effect” does not always hold, and it is affected by various factors. For example, the influence of “Pygmalion effect” on female leaders is less than that of male leaders; it also has less influence on existing subordinate groups than newcomers (White and Locke, 2000). In recent years, researchers have begun to focus on the bidirectional nature of social support. There are indeed advantages to both giving and accepting support (Du et al., 2016).

Role indicates the expectations and desires of the individual and organization from each other (Adil et al., 2021). Hence, considering only the role expectations of employees set by their supervisors does not fully reflect the potential for innovation. Employees’ cognition of their role (namely, their role clarity) may play the same critical role in the effects of leader creativity expectations. As creativity cannot be separated from the specific content of an employee’s work or the environment in which that work is performed (Baer, 2003). Role clarity indicates the extent to which employees acquire and understand the information or data required to complete their work (Kelly and Richard, 1980; Adil et al., 2021). The novelty, complexity, and ambiguity of problems differ among the different people who solve them and the problems that require creative thinking in a certain environment may not translate directly to other environments. Objectives, responsibilities, and rules of behavior also may differ depending on the employee’s position, which affect his or her role clarity (Stinglhamber and Vandenberghe, 2004; Panaccio and Vandenberghe, 2011; Orgambídez et al., 2022).

Role clarity, as mentioned above, refers to the extent to which employees believe they have clear guidance for the expected behavior relevant to their job (Jada et al., 2019). Intuitively, role clarity gives employees clear expectations for their performance. Studies have shown that the employees with high role clarity work under an appropriate amount of pressure (Gilboa et al., 2008), less physical fatigue and psychological discomfort (Cenzig et al., 2021; Orgambídez et al., 2022), and have relatively high psychological empowerment (Hall, 2008), which support them in conducting work-related activities independently and creatively. Employees with role clarity report a stronger sense of support from their leaders, which encourages them to take their duties more seriously (Eisenberger et al., 1990; Stinglhamber and Vandenberghe, 2004; Newman et al., 2015). Employees with higher role clarity also tend to have the resources and psychological support necessary to explore and innovate within the parameters of their work responsibilities (Gkorezis, 2016). Employees with low role clarity tend to feel more stress and anxiety at work; an ambiguous environment leaves individuals unable to understand the expectations of the company or leaders regarding their performance (Newman et al., 2015), which is also fed back into the organization and can weaken the effects of any existing leader creativity expectations.

For employees with proactive personalities who perform innovative activities, timely feedback between their leaders’ expectations of creativity and role clarity can further strengthen the connection between proactive personality and creativity. We propose the following hypothesis:

*H3*: The interaction between role clarity and leader creativity expectations influences the relationship between proactive personality and employee creativity; the positive relationship between proactive personality and employee creativity is strongest when both role clarity and leader creativity expectations are high.

## Materials and methods

### Participants

We issued questionnaires to the innovation teams of several subsidiaries of China XD Group in early December 2020. As one of the largest state-owned enterprises in China, XD Group’s business includes real estate development, new materials engineering, and healthcare. There were relatively active levels of formal and informal exchanges among members of the group. Therefore, supervisors were able to easily obtain information about subordinate actions, and every supervisor who had the opportunity to observe their employees’ creative behavior was invited to finish the scoring task. Most of the respondents (87.2%) are engaged in new product development, with only a small number of human resources personnel and manager supporters. Therefore, the sample is suitable for hypothesis testing and the selection bias in this study is low. Table 1 presents the relevant information of participants.

**Table 1:** Means, standard deviations, and correlations.

Variables	Mean	SD	1	2	3	4	5	6	7	8	9
1 Employee age	26.86	3.236									
2 Gender	0.60	0.491	−0.009								
3 Tenure	2.02	0.834	0.676^**^	0.076							
4 Job type	0.37	0.483	−0.081	0.498^**^	−0.059						
5 Prosocial motivation	3.01	0.860	−0.003	−0.020	0.014	0.013					
6 Proactive personality	3.16	0.782	−0.022	0.113	0.028	0.083	−0.055	(0.76)			
7 Leader creativity expectations	2.98	0.870	0.005	−0.016	−0.114	0.040	0.037	0.029	(0.75)		
8 Role clarity	3.09	0.979	0.015	0.109	0.028	0.159^**^	0.023	0.470^**^	−0.144^*^	(0.84)	
9 Employee creativity	3.26	0.898	0.008	−0.025	−0.001	0.061	0.117^*^	0.317^**^	0.174^**^	0.207^**^	(0.84)

*N* = 290.

* *p <* 0.05 and

** *p <* 0.01.

AVE values are on the diagonal in parentheses.

To prevent common method bias to the maximum extent, we adopted a matching “supervisor-subordinate” sample. The respondents were ordinary employees and their direct supervisors within the company. The leader provided corresponding evaluations (A) to the direct subordinate regarding creativity. The employee evaluated his or her leader’s creativity expectations, their own proactive personalities, and their role clarity (B) All participants provided personal information in completing the questionnaire, which was kept confidential. A total of 565 pairs of questionnaires were issued in the survey, among which 290 “supervisor-subordinate” pairs were matched. The overall effective recovery rate of the questionnaires was 51.33%. The reason for the low response rate may be that people in the Confucian cultural background characteristics by collectivism and high power distance are unwilling to express their views easily in many cases, especially employees from state-owned enterprises.

Among them, male employees account for 59.7% and female employees for 40.3%. The overall age structure of employees is relatively young, with a minimum age of 21 years and a maximum of 35 years. Up to 73.1% of the total, 212 of the employees, hold a Bachelor’s degree; 136 have Master’s degrees, accounting for 46.9% of the total; and 17 employees (5.9%) hold PhDs. Leaders and employees had worked together for an average of 3.25 years at the time of their participation, so the data obtained from the questionnaire can be regarded as based on a mutual understanding between them. The employees are considered to have a relatively clear understanding of their own abilities with a certain level of objectivity and accuracy.

### Procedure

Before administering the questionnaire, we informed all department heads, supervisors, and volunteers of the purpose and process of this study, and of their privacy rights in participating. Our coordinator gave respondents detailed instructions on the procedures for completing the investigation and the purpose of the study. Additionally, we attached a survey description to each questionnaire that guaranteed the confidentiality of our investigation, with the corresponding certificate number of the supervisor and subordinates displayed in advance in order to match the reply of each interviewee. Furthermore, we prepared a small gift for all participants. All procedures performed in studies involving human participants were in accordance with the ethical standards of the institutional and/or national research committee and with the 1964 Helsinki Declaration and its later amendments.

Considering the complexity of paired sampling and the sensitivity of mutual ratings, we have carefully designed the steps of the research and prepared the materials that need to be used. To avoid common method biases and potential biases, we made a separate questionnaire for each subordinate and their direct supervisor. We also used the upper and lower pairing method to obtain relevant data, distributed it to upper and lower staff, and asked each supervisor to complete a questionnaire for only one subordinate. Specifically, the team leader completes the leadership questionnaire, evaluates the creativity of their direct subordinate, and fills in the personal information. The direct subordinate of the team leader then completes the employee questionnaire (including the proactive personality and leader creativity expectations, role clarity), and fills in the personal information.

The scales were translated and re-translated by three doctoral students familiar with both Chinese and English (Brislin, 1980). All items were measured using the Likert 5-point scale (from 1 = strongly disagree or not at all, to 5 = strongly agree or a great deal). In the first wave, with the assistance of department heads and supervisors, we invited employees to participate in answering the corresponding questions. In the second wave, we invited department heads/supervisors to complete the evaluation of employee creativity.

These samples are divided into two groups according to the type of industry using to the method proposed by Frazier et al. (2009) for purpose of examining the non-response bias. The *t*-test results of these two different groups’ samples showed no significant difference. Therefore, non-response bias is not a factor that needs to be particularly concerned in this study. In addition, Harman’s one-factor test is also be applied to examine common method bias in our study (Frazier et al., 2009). The results show that the first principal component explains for 36.58% of the variance, demonstrating that no single factor exists to account for most of the variance, which further signifies that the common method bias is not serious.

### Measure

The scale used in this study was adapted from international mainstream journals, please refer to the Appendix for details. We followed the standard procedure of literal translation and back-translation to ensure that each item’s content was accurately maintained after translation. We used a five-level Likert scale to measure the items, where “1” represents “strongly disagree” and “5” represents “strongly agree.”

#### Proactive personality

We measured the employees’ proactive personality on 10 items developed by Seibert et al. (1999), including “I have been looking for new ways to improve [my] life,” “no matter where I am, I am always the important force to make constructive changes.” Cronbach’s α for this scale was 0.97.

#### Employee creativity

We measured employee creativity level on the four items developed by Farmer et al. (2003), including “[I] will first try new ideas or methods” and “[I] will find new… methods when solving problems.” The Cronbach’s α value is 0.96.

#### Leader creativity expectations

We measured leader creativity expectations on the four items developed by Carmeli and Schaubroeck (2007). The employees reported their perceived expectations of their direct subordinate leaders for innovation, including “My direct [superior] expects me to be creative at work” and “My direct [superior] expects me to creatively finish [my] work.” The Cronbach’s α value is 0.92.

#### Role clarity

We measured role clarity based on a five-item scale from Rizzo et al. (1970) sample items included “I feel certain about how much authority I have” and “There are clear, planned goals and objectives for my job.” The Cronbach’s α value is 0.96.

#### Control variables

We control for variables including age, gender, tenure, and job type, which have been found to be significantly related to employee creative performance (Zhang and Bartol, 2010; Harris et al., 2014). Specifically, age is measured in years. Gender is manipulated as a dichotomous variable coded as 0 for females and 1 for males. Tenure is measured as the number of years that an employee had been with an enterprise (Code: 1 for “< 1 year,” 2 for “1 to <3 years,” 3 for “3 to <10 years,” and 4 for “10 to <20 years”). Job type is also manipulated as a dichotomous variable, where 0 represents employees working in R&D departments and 1 represents employees working in non-R&D departments (such as employees working in strategic marketing and functional departments). Furthermore, we control employees’ prosocial motivation since employees with highly prosocial behavior may spontaneously engage in creative actions within norms. The scale consists of four items, and a sample item is “I care about benefiting others through my work.” The Cronbach’s α value is 0.93.

## Results

**Figure 2:**
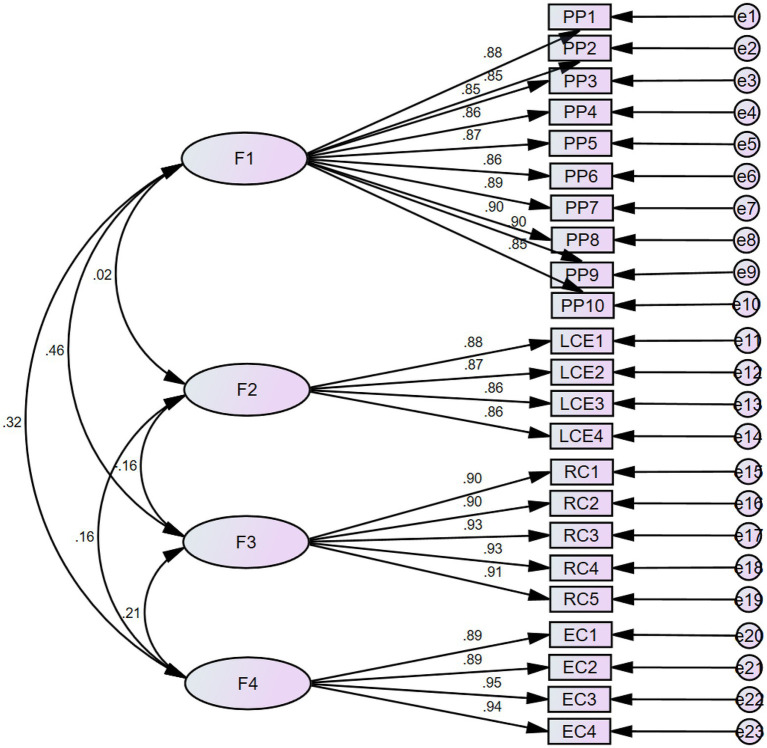
Confirmatory factor analysis.

Therefore testing our model, we first analyzed the reliability and validity of the scale. The Cronbach’s Alpha is greater than 0.7 with the factor load over 0.7 and AVE over 0.6, indicating that the scale has good reliability and validity. The proposed four-factor model (proactive personality, leader creativity expectations, role clarity, and employee creativity) exhibited an adequate fit with the data (χ^2^/df = 1.986, χ^2^ = 444.929, df = 224, CFI = 0.969, NFI = 0.940, RMSEA = 0.058). The mean value, standard deviation, correlativity values, and AVE values, as shown in Table 1, indicate that all the major variables are significantly correlated with employee creativity. The results of confirmatory factor analysis are shown in Figure 2. The correlation among variables provides preliminary support for verifying our hypotheses.

To verify our hypotheses more exactly, we conducted two-step regression analysis by SPSS PROCESS 3.3 through Model 1 and Model 3. The results are showed in Tables 2 and 3. As shown in Table 2, we can see that the regression coefficient of proactive personality and employee creativity is 0.368 (*p* < 0.001), namely, proactive personality has a positive and significant effect on employee creativity, thus H1 was supported.

**Table 2:** Hierarchical regression results of Model 1.

	Standardized coefficient	SE	*t*	*p*	95%CI	
					LL	UL
Outcome variable: employee creativity						
constant	2.723	0.507	5.367	0.000	1.724	3.722
Proactive personality	0.368	0.064	5.782	0.000	0.243	0.494
Leader creativity expectations	0.166	0.058	2.878	0.004	0.053	0.279
Int_1	−0.060	0.064	−0.931	0.353	−0.186	0.067
Employee age	0.004	0.021	0.209	0.835	−0.037	0.045
Gender	−0.163	0.117	−1.387	0.166	−0.394	0.068
Tenure	0.016	0.082	0.200	0.842	−0.145	0.178
Job type	0.144	0.119	1.219	0.224	−0.089	0.378
Prosocial motivation	0.142	0.058	2.456	0.015	0.028	0.255
Model summary						
*R*	*R* ^2^	MSE	*F*	df_1_	df_2_	*p*
0.3963	0.157	0.699	6.544	8	281	0.000

*N =* 290; Product terms key: Int_1: Proactive personality x Leader creativity expectations.

**Table 3:** Hierarchical regression results of Model 3.

	Standardized coefficient	SE	*t*	*p*	95%CI	
					LL	UL
Outcome variable: employee creativity						
constant	2.801	0.488	5.736	0.000	1.840	3.763
Proactive personality	0.331	0.071	4.666	0.000	0.191	0.471
Leader creativity expectations	0.116	0.062	1.865	0.063	−0.007	0.238
Int_1	−0.098	0.072	−1.361	0.175	−0.239	0.044
Role clarity	0.042	0.058	0.718	0.474	−0.073	0.156
Int_2	0.141	0.050	2.843	0.0048	0.043	0.238
Int_3	0.101	0.054	1.859	0.064	−0.006	0.208
Int_4	0.148	0.044	3.392	0.0008	0.062	0.234
Employee age	−0.002	0.020	−0.108	0.914	−0.042	0.037
Gender	−0.165	0.113	−1.466	0.144	−0.387	0.057
Tenure	0.041	0.079	0.514	0.608	−0.115	0.196
Job type	0.127	0.115	1.103	0.271	−0.100	0.353
Prosocial motivation	0.150	0.056	2.704	0.007	0.041	0.259
Conditional effects of the focal predictor at values of the moderator(s)						
Leader creativity expectations	Role clarity	Effect	SE	t	*p*	
−0.870	−0.979	0.405	0.124	3.268	0.0012	
−0.870	0.000	0.416	0.096	4.331	0.0000	
−0.870	0.979	0.428	0.104	4.129	0.0000	
0.000	−0.979	0.193	0.092	2.112	0.0356	
0.000	0.000	0.331	0.071	4.666	0.0000	
0.000	0.979	0.469	0.080	5.866	0.0000	
0.870	−0.979	−0.018	0.108	−0.170	0.8655	
0.870	0.000	0.246	0.093	2.641	0.0087	
0.870	0.979	0.510	0.114	4.455	0.0000	
Model summary						
R	R^2^	MSE	F	df_1_	df_2_	p
0.485	0.235	0.644	7.103	12	277	0.000

*N* = 290; Product terms key: Int_1: Proactive personality × Leader creativity expectations; Int_2: Proactive personality × Role clarity; Int_3: Leader creativity expectations × Role clarity; and Int_4: Proactive personality × Leader creativity expectations × Role clarity.

We next verified H2. As the results shown in Table 2, the regression coefficient of the interaction term on employee creativity between proactive personality and leader creativity expectations is −0.060 (*p* > 0.1), namely, leader creativity expectations do not significantly moderate the relationship between proactive personality and employee creativity. Higher expectations do not strengthen the relationship over lower expectations, so H2 was not supported.

**Figure 3:**
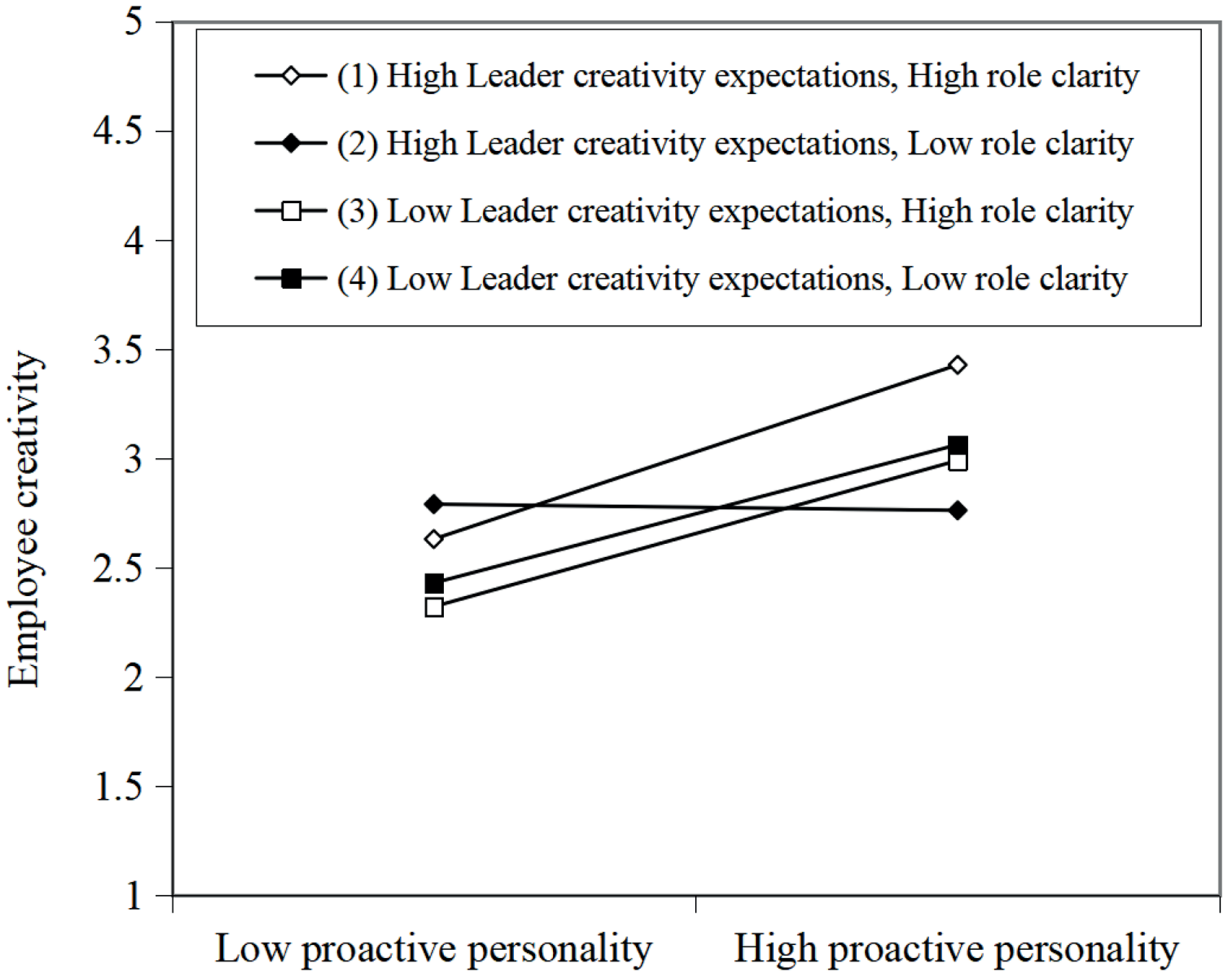
Three-way interaction effects of proactive personality, leader creativity expectations, and role clarity on employee creativity.

Table 3 shows that the interaction between leader creativity expectations and role clarity has a significant moderating effect on the relationship between proactive personality and employee creativity (β = 0.148, *p* < 0.001). Besides, the results of conditional effects of the focal predictor at values of the moderator(s) show that when both role clarity and leader creativity expectations are high (M + 1SD), the positive correlation between proactive personality and creativity is strongest (β = 0.510, *p* < 0.001). Referring to Cohen et al. (2013), we plot the simple slopes to uncover the nature of the significant three-way interactions by conventional procedures for high (M + 1SD) and low (M-1SD) focal variables. Figure 3 clearly shows that the significant three-way interaction effects of proactive personality, leader creativity expectations, and role clarity on follower creativity. As shown in Figure 3, the positive relationship between proactive personality and employee creativity is significantly proven for high leader creativity expectations—high role clarity (slope 1, β = 0.510, *p* < 0.001) and high leader creativity expectations—low role clarity (slope 2, β = −0.018, *p* > 0.1), while the relationships between proactive personality and employee creativity are still significantly proven for low leader creativity expectations—high role clarity (slope 3, β = 0.428, *p* < 0.001) and low leader creativity expectations—low role clarity (slope 4, β = 0.405, *p* < 0.01), which means that proactive personality has the most positive effect on employee creativity only when both leader creativity expectations and role clarity are high. Hence, H3 was supported.

## Discussion

This study examined the interactive role of synergistic interplay among proactive personality, leader creativity expectations, and role clarity on employee creativity. We investigated the joint moderating effect between leader creativity expectations and role clarity to reveal the function of role shaping in the process of creative problem-solving as affected by personal proactive personality.

We did find a positive correlation between proactive personality and creativity; however, we did not find that the leader creativity expectations moderate the relationship between proactive personality and employee creativity. Though proactive personality is a key factor in enhancing intrinsic motivation (Horng et al., 2016; Mubarak et al., 2021), the process of activating such motivation may be influenced by the external environment (Joo and Lim, 2009; Liao, 2022). According to self-determination theory, once an individual feels that he or she is influenced by external circumstances to take action, any “autonomous” motivations otherwise felt will be significantly reduced. Extremely strong leader creativity expectations may be perceived as a job requirement (Montag et al., 2012; Zhao and Guo, 2019), in which case employees may perform creative behaviors at the behest of their supervisors rather than autonomously (Shin et al., 2017). Creativity expectations can thus be regarded as a form of controlled motivation (Gagné and Deci, 2005).

Controlled motivation refers to the motivation of an individual beyond his or her voluntary will or free choices to engage in a certain behavior under internal (e.g., guilt) or external (e.g., demands of others) pressure. The degree of autonomy over an individual’s behavior is relatively weak. Some researchers believe that controlled motivation functions negatively in terms of hindering individual behavior (Deci and Ryan, 2000; Baumeister and Vohs, 2007). Grant et al. (2011) further pointed out that the co-occurrence of autonomic motivation and controlled motivation may result in ineffective behaviors. Namely, controlled motivation may inhibit creativity in work situations or other situations that require innovation. When leader creativity expectations are regarded as controlled motivation, the employee may lose his or her enthusiasm for otherwise highly autonomous and proactive behaviors (Zhao and Guo, 2019).

After considering the individual contingency factor of role clarity, we found that the interaction between leader creativity expectations and role clarity significantly enhances the above relationship, playing a positive moderating role between proactive personality and employee creativity. It is possible that when facing even very high leader creativity expectations, as long as the employee has strong role clarity, he or she can effectively prioritize creativity in regard to the current responsibilities and scope of his or her position. In such cases, leader creativity expectations may not be regarded as a type of controlled motivation as innovation is a “responsibility” rather than “additional work.” Thus, employees with strong proactive personalities are more likely to show higher creativity when the creative activities are regarded as a part of their job.

### Theoretical significance

We identified a significant positive role of synergistic interplay among proactive personality, leader creativity expectations, and role clarity in stimulating employee creativity, which may enrich relevant research on the Pygmalion effect of creativity. Previous studies have shown that proactive personality can perform as a key antecedent variable for employee creativity (Tai and Mai, 2016; Kim, 2019; Wang et al., 2022; Zhang et al., 2022), and this study further confirms the relationship between proactive personality and employee creativity. This result may be due to the great emphasis that Confucian societies place collectivism. Confucian collectivism urges people to sacrifice individual interests for collective interests in many cases, although the changes brought by proactive personality will challenge the established work objectives, working methods, task relationships, and informal norms (Bysted and Jespersen, 2014).

Secondly, this research helps deepen our understanding of the Pygmalion effect of creativity. Even for employees with proactive personalities, leader creativity expectations may not necessarily be effective in enhancing employee creative performance. This result supports the finding of Zhao and Guo (2019) to a certain extent, and also indicates that employee with proactive personality has stronger intrinsic motivation rather than controlled motivation (Ng and Feldman, 2013; Horng et al., 2016; Kim, 2019; Mubarak et al., 2021). Besides, we found that although leader creativity expectations do not significantly enhance the relationship between proactive personality and employee creativity, once role clarity (a “contingency factor”) is considered, the interaction between leader creativity expectations and role clarity does positively moderate the relationship between proactive personality and employee creativity. Under the conditions of both extremely high leader creativity expectations and role clarity, there is a strong positive correlation between proactive personality and creativity. This finding enriches our empirical understanding of the connotations of role shaping. According to the role theory (Anglin et al., 2022), role shaping should not only rely on leader role expectations, but more importantly, employees’ own role cognition and role-related learning. Role clarity indicates the extent to which employees acquire and understand the information or data required to complete their work (Kelly and Richard, 1980; Adil et al., 2021). Role clarity can create “mutual matching” between individual factors and contextual factors. Shalley et al. (2004) also found that matching between individual personality characteristics and situations can make employees more creative, which supports the conclusions we reached in the present study.

### Practical significance

Our results may have significance in terms of managerial practices. Organizations may enhance their overall creativity by selecting employees with specific personality traits (Zhang et al., 2020). Proactive employees are relatively more creative, so hiring or promoting those with stronger proactive personalities may be useful—especially for positions that explicitly require creative problem-solving. Organizations should also understand the key role of leaders, particularly in regard to establishing and enforcing creativity expectations as well as providing role clarity. Leader creativity expectations at a certain level can damage the sense of self-determination in employees with stronger proactive personalities, thereby damaging their creativity, so administrators should dynamically adjust the expectations assigned to different types of employees’ creative behaviors. The organization should help employees to clearly understand their roles, clearly communicate the specific duties of their positions and relevant tasks, strengthen job training specific to certain roles, and encourage leadership. The results of this study also prove that mutual matching between individual factors and situational factors stimulates employee creativity to the greatest extent possible. When an organization intends to stimulate the overall creativity, it would benefit from matching appropriate team leaders to employees based on their individual personal characteristics.

### Limitations and future research

Although the vector relationships explored in our hypotheses are consistent with previous studies, cross-sectional data did not serve as an ideal design for establishing the causal order between the proposed relationships. Future work may use multi-wave time-lagged research to provide more accurate inferences. The second limitation of the study is that role clarity is in fact “perceived role clarity” as the actual role clarity is not measured, only the perceived. Future research can adopt indicators of specific work demands that can objectively reflect the level of role clarity. Third, we did not examine the possible mediating effects between the three-way interaction and employee creativity. It is of great significance to investigate the mediating mechanism, as it will help reveal the method by which proactive personality is related to employee creativity.

## Data availability statement

The raw data supporting the conclusions of this article will be made available by the authors, without undue reservation.

## Ethics statement

Ethical review and approval was not required for the study on human participants in accordance with the local legislation and institutional requirements. The patients/participants provided their written informed consent to participate in this study.

## Author contributions

XW, MW, and FX jointly designed the general idea and outline of the manuscript. FX and XW created a first draft. FX and MW revised the manuscript. All authors contributed to the article and approved the submitted version.

## Funding

This research was supported by the National Natural Science Foundation of China (nos. 72102056 and 71874042), Philosophy and Social Science Program of Heilongjiang (no. 21EDE318), Postdoctoral Sustentation Fund of Heilongjiang Human Resources and Social Security Bureau (no. LBH-Z19148), and Fundamental Research Funds for the Central Universities (no. HIT.HSS.202125).

## Conflict of interest

The authors declare that the research was conducted in the absence of any commercial or financial relationships that could be construed as a potential conflict of interest.

## Publisher’s note

All claims expressed in this article are solely those of the authors and do not necessarily represent those of their affiliated organizations, or those of the publisher, the editors and the reviewers. Any product that may be evaluated in this article, or claim that may be made by its manufacturer, is not guaranteed or endorsed by the publisher.

## Appendix

Proactive personality

I have been looking for new ways to improve [my] life.

No matter where I am, I am always the important force to make constructive changes.

Nothing is more exciting than seeing my ideas turn into reality.

If I see something I do not like, I fix it.

No matter what the odds, if I believe in something I will make it happen.

I love being a champion for my ideas, even against others’ opposition.

I excel at identifying opportunities.

I am always looking for better ways to do things.

If I believe in an idea, no obstacle will prevent me from making it happen.

I can spot a good opportunity long before others can.

Employee creativity

I will first try new ideas or methods.

I will find new ideas and methods when solving problems.

I will Generates ground-breaking ideas related to the field.

I am a good role model for creativity.

Leader creativity expectations

My direct [superior] expects me to creatively finish [my] work.

My supervisor thinks that creativity is important to me.

My direct [superior] expects me to be creative at work.

My supervisor would probably be disappointed in me if I was not creative.

Role clarity

I feel certain about how much authority I have.

There are clear, planned goals and objectives for my job.

I know exactly what is expected of me.

I know what my responsibilities are.

I know that I have divided my time properly.
